# Development of a Chemiluminescence Immunoassay for Serum YB-1 and its Clinical Application as a Potential Diagnostic Marker for Hepatocellular Carcinoma

**DOI:** 10.5812/hepatmon.8918

**Published:** 2013-07-03

**Authors:** Li Pu, Shi Jing, Guo Bianqin, Liu Ping, Liang Qindong, Liu Chenggui, Cheng Feng, Kuang Wenbin, Wang Qin, Dong Jinyu, Xia Qianfeng, Liu Yu, Tu Zhiguang

**Affiliations:** 1 Key Laboratory of Clinical Laboratory Diagnostics, College of Laboratory Medicine, Chongqing Medical University, Chongqing, China; 2 Department of Clinical Laboratory, The First Affiliated Hospital, Chongqing Medical University, Chongqing, China; 3 Department of Laboratory Medicine, Hainan Medical College, Hainan, China; 4 Department of Clinical Laboratory, Chongqing Tumor Hospital, Chongqing, China

**Keywords:** Y-Box-Binding Protein 1, Carcinoma, Hepatocellular, Tumor Marker

## Abstract

**Background:**

Y-box binding protein 1 (YB-1) overexpression has been shown in various tumor cells including hepatocellular carcinoma (HCC); moreover, this protein can be actively secreted.

**Objectives:**

The aim of this study was to establish a method to quantify serum YB-1 and evaluate its clinical application in the clinical diagnosis of HCC.

**Patients and Methods:**

Recombinant YB-1 and two populations of its antibodies were prepared. A monoclonal antibody was specific to the N-terminus of YB-1 amino acids 134-160; and another was a polyclonal antibody. A sandwich-type chemiluminescence immunoassay (CLIA) was developed and evaluated. Levels of YB-1 and alpha fetoprotein (AFP) in serum samples from 105 HCC patients, 25 hepatitis B virus patients, 25 cirrhosis patients, and 50 healthy donors were detected using the established method and an AFP electrochemiluminescence kit.

**Results:**

The developed method was linear to 150 μg/L of YB-1 with a minimum detection limit of 0.01 μg/L. The average recoveries were between 93.9% and 109.0%. The mean intra- and inter-assay coefficients of variation (CVs) were 4.0-4.8% and 8.2-10.2%, respectively. The relationship between the concentration of diluted YB-1 and the dilution ratios gave a good linear correlation coefficient of 0.9986. The YB-1 concentration was increased in serum of HCC patients (33.0 ± 23.39 μg/L) compared to healthy individuals (13.2 ± 5.29 μg/L, P < 0.0001), patients with HBV (17.9 ± 7.49 μg/L, P = 0.0003), and patients with HBV cirrhosis (20.7 ± 8.75 μg/L, P < 0.05). Moreover, the combination of YB-1 and alpha-fetoprotein had a high sensitivity (89.5%) and reasonable specificity (62.0%) in identifying HCC.

**Conclusions:**

The established method has an acceptable performance in quantifying YB-1. In addition, serum YB-1 may aid in the diagnosis of HCC.

## 1. Background

Hepatocellular carcinoma (HCC) is one of the lethal forms of malignant tumors with poor prognosis and difficult for early diagnosis due to the lack of reliable biomarkers. Alpha-fetoprotein (AFP), widely used for diagnosis and surveillance, shows a low diagnostic sensitivity for HCC (1). Other recently discovered markers as promising tools for HCC diagnosis, include abnormal prothromhin (APT), lens culinaris agglutinin-reactive AFP (AFP-L3) and dickkopf-1 (2, 3). These markers, however, are only used for screening HCC with AFP-negative and are still under research. Y-box binding protein 1 (YB-1), an oncogenic transcription/translation factor, belonging to the highly conserved cold-shock domain protein superfamily, plays pleiotropic functions in the cell cycle, such as the regulation of transcription and translation (4). YB-1 is overexpressed in many malignant cells (5, 6). Considerable research has been conducted regarding the role of intracellular elevated levels of YB-1 mRNA and protein by RT-PCR and Western blotting in cancer progression and chemotherapy resistance. Immunohistochemical observations have demonstrated that overexpression of YB-1 occurs in a variety of cancer cells and tissues, and is involved in tumorigenesis, tumor growth, and disease progression (7-9). The cytoplasmic and predominant nuclear localization of YB-1 is consistent with the intrinsic expression of many oncogenes (7, 9-13), response to oxidative stress, and coordination of DNA excision repair (14), and has been associated with an unfavorable outcome in cancer patients (13, 15, 16). A recent study confirmed that YB-1 can be actively secreted through a non-classical pathway in the presence of cytokines and oxidative stress (17). Furthermore, Tacke (18) reported that the active secretion of YB-1 in plasma can be detected by Western blotting, and a fragment of YB-1 (18kDa) was identified as an independent biomarker for patients with malignancy diseases. Investigating the significance of serum YB-1 levels in cancer patients is limited due to a lack of a suitable sensitive and specific quantitative detection method, which could potentially allows diversification of the relative risk profile and sensitivity to chemotherapy (12, 19).

## 2. Objectives

In the present study, recombinant YB-1 protein and specific YB-1 monoclonal (mAbs) and polyclonal antibodies (pAbs) were prepared. A double antibody sandwich chemiluminescence immunoassay (CLIA) method was developed to detect serum YB-1 and applied for clinical diagnosis. Moreover, the diagnostic sensitivity, specificity, and accuracy of serum YB-1 for HCC were evaluated and compared with AFP and the combination of YB-1 and alpha-fetoprotein (AFP).

## 3. Patients and Methods

### 3.1. Reagents, Cell strains, and Apparatus

Female BALB/c mice were purchased from the Animal Center of Chongqing Medical University (Chongqing, China). The origin of the myeloma cell line, SP2/0, was from the Institute of Biochemistry and Cell Biology (Chinese Academy of Sciences, Shanghai, China). The renal cancer cell line, 786-0, the breast cancer cell line, MDA-MB-231, the HCC cell line, HepG2, and the non-small cell lung cancer (NSCLC) cell line, A549, were obtained from our laboratory. Goat anti-mouse IgG-horseradish peroxidase (HRP) and the Rapid ELISA Mouse mAb Isotyping Kit were obtained from Pierce (Rockford, IL, USA). Rabbit mAb to YB-1 (ab76540; Abcam, Cambridge, MA, USA). All other reagents were of analytical grade. All washing steps were carried out with an automatic plate washer (Bioscience, Tian Jin, China). The PETECK96-II Detection System (Bioscience) was used to read relative light units (RLU) at 450 nm. AFP was detected using an ARCHITECT i2000sr electrochemiluminescence analyzer with an Architect AFP kit (Abbott Diagnostics, Abbott Park, IL, USA).

### 3.2. Study Population and Sample Collection

In the current study, we determined the serum YB-1 concentrations of 41 female and 64 male patients between 40 and 65 years of age (mean, 53.8 years; median, 54 years) with various stages of HCC (Table 1).

**Table 1.** Demographic and HCC Characteristics

HCC characteristics	No. (%)
**Overall patients**	105
**Tumor status**	
T1	10 (10)
T2	31 (30)
T3	50 (47)
T4	14 (13)
**Nodal status**	
N1	24 (23)
N2	54 (51)
N3	27 (26)
**Metastasis**	
M0	63 (60)
M1	42 (40)
**Stage**	
1	10 (10)
2	35 (33)
3	43 (41)
4	17 (16)

We also determined the serum YB-1 levels of 25 age- and gender-matched hepatitis B virus (HBV)-infected patients, 25 HBV patients with advanced stage cirrhosis diagnosed according to the results of histopathological examinations or the combined results of clinical, laboratory, and imaging examinations, and 50 healthy volunteers. All of the HCC patients were diagnosed based on histopathological findings. These subjects were all recruited from the Chongqing Tumor Hospital in China, and all subjects gave written informed consent to participate in the study. The study was approved by the Science and Ethic Committee of Chongqing Medical University and was performed in accordance with the Declaration of Helsinki. Measurement of serum YB-1 and AFP was performed in HCC patients before treatment and surgery and all control subjects. Blood samples were collected in vacuum blood collection tubes and centrifuged at 2500 x g for 10 min at 4°C within 30 min after the formation of the blood clot. The sera aliquots were stored at -80°C until analyzed.

### 3.3. Recombinant Human YB-1 Protein Preparation

#### 3.3.1. Amplification of the YB-1 Gene by RT-PCR

The YB-1 cDNA coding sequence (GenBank ID: NM_004559.3) was obtained by RT-PCR using the total RNA from the human renal cancer cell line, 786-0, as a template with the following primers: ATGAGCAGCGAGGCCGAGAC; and TTACTCAGCCCCGCCCTGCT. The sequence was cloned into vector pGEX-6P-1 (Invitrogen, Carlsbad, CA, USA) and confirmed by DNA sequencing.

#### 3.3.2. Expression and Purification of Recombinant YB-1

For prokaryotic expression and purification of human YB-1 (20), the recombinant plasmid was transformed into Escherichia coli strain BL21 (DE3; Novagen, Madison, WI, USA), and induced by the addition of 0.5 mM isopropyl-β-D-thiogalactoside at 20℃ for 6 h. To purify the well-expressed fusion protein, 1.0 g of cultured cells were lysed on ice by ultrasonication and subjected to centrifugation at 20 000 x g at 4°C for 30 min. The supernatants, then underwent glutathione S-transferase tag (GST-tag) affinity chromatography (Thermo Scientific, Waltham, MA, USA) according to the manufacturer’s instructions. Removal of the GST-tag from the affinity-purified fusion protein was achieved by incubating 20 μg of the fusion protein with 1 unit of PreScission Protease (PSP; GE Healthcare, Piscataway, NJ, USA) in PBS and 1 mmol/L DTT at 4°C for 24 h, and further purification was achieved by ion exchange chromatography using an ÄKTApurifier^TM^ Versatile FPLC purification system (GE Healthcare) to obtain highly purified YB-1 protein (≥ 95%). Ultrafiltration concentration of the purified YB-1 from column eluents was performed with an Amicon Ultra-15 Centrifugal Filter Unit (Millipore, Billerica, MA, USA). The final cleaved product was analyzed by SDS-PAGE and Western blot.

### 3.4. Preparation of Antibodies

Rabbit anti-human YB-1 pAbs were prepared by injecting a rabbit subcutaneously 4 times at 2-week intervals with 2 mg of recombinant human YB-1 emulsified with an equal volume of Freund’s adjuvant. The IgG fraction of antiserum was purified by protein A sepharose column chromatography (GE Healthcare). Purified pAbs were conjugated with HRP using an EZ-Link Plus Activated Peroxidase Kit (Pierce) according to the manufacturer’s coupling protocols. YB-1 mAb was prepared by immunizing mice with 100 μg of recombinant YB-1 emulsified with an equal volume of complete Freund’s adjuvant and subcutaneously injected into female BALB/c mice 4 times at 2-week intervals. Four days after the last booster injection, spleen cells obtained from the immunized mice were fused with mouse myeloma cells (Sp2/0) in the presence of 50% polyethylene glycol and 7.5% DMSO (21), and cultured according to standard procedures. Hybridoma culture supernatants from 96-well plates were screened using an ELISA with recombinant YB-1 protein as the specific antigen. The positive-detected hybridoma cells were single-cells cloned five times by limited dilution. A clone was selected to yield a mAb (1-D9), which could specifically recognize YB-1 based on Western blot analysis. Ascites immunoglobulins, which were induced by injecting the 1-D9 hybridoma cells (0.5 × 10^6^) into the peritoneal cavity of BALB/c mice, were purified with protein G sepharose columns (GE Healthcare). The isotype of YB-1 mAb was identified using a Rapid ELISA Mouse mAb Isotyping Kit (Pierce) according to the manufacturer’s instructions.

### 3.5. Epitope Identification

**Figure 1.**
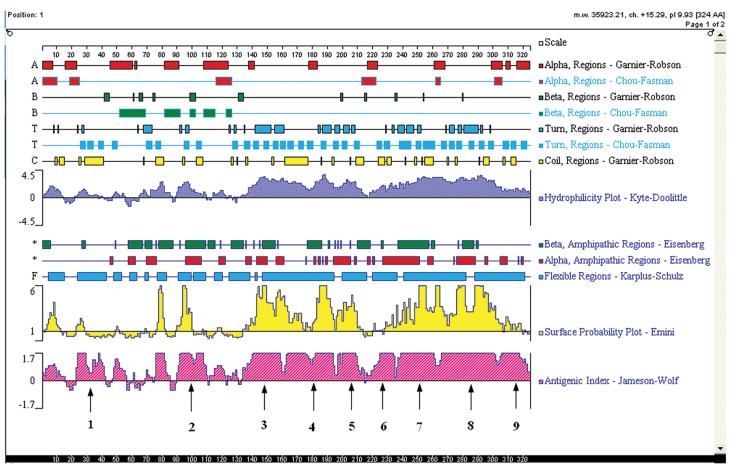
The Linear Epitopes of YB-1 Protein Predicted by DNAstar Arrows indicate the linear epitopes of YB-1 protein

The sequence of YB-1 protein (GenBank ID: NP-004550.2) was analyzed using the DNAstar program protean (DNAstar, Madison, WI, USA). According to the predicted results of DNAstar, YB-1 proteins probably exist as 9 linear epitopes as follows: 1 (18-42aa); 2 (90-110aa); 3 (134-160aa); 4 (162-195aa); 5 (197-215aa); 6 (216-235aa); 7 (236-265aa); 8 (266-303aa); and 9 (304-324aa; Figure 1). The truncated genes (1-9) of YB-1 were obtained using the recombinant vector pGEX/YB1 as a template with the PCR primers shown in Table 2.

**Table 2.** Primers for Lopping DNA Sequences of YB-1 a

Numbers 1-9	Primers (5’-3’)
**1 (18-42aa)**	F:GGATCCATGCCCGCCCTCAGCGCC
	R:CTCGAGTTAGAGGCCGCCCGGGCC
**2 (90-110aa)**	F:GGATCCATGGCCATAAAGAAGAAT
	R:CTCGAGTTACTCCACAGTCTCTCC
**3 (134-160aa)**	F:GGATCCATGCAAGGCAGTAAATAT
	R:CTCGAGTTATTGCTGGTAATTGCG
**4 (162-195aa)**	F:GGATCCATGTACCAGAATAGTGAG
	R:CTCGAGTTAAGGTGGGAACCTTCG
**5 (197-215aa)**	F:GGATCCATGTACATGCGGAGACCC
	R:CTCGAGTTATCCCTGCACAGGAGG
**6 (216-235aa)**	F:GGATCCATGGAAGTGATGGAGGGT
	R:CTCGAGTTACTGCCTCACTGGTCT
**7 (236-265aa)**	F:GGATCCATGAATATGTATCGGGGA
	R:CTCGAGTTATTCTTTATCTTCTTC
**8 (266-303aa)**	F:GGATCCATGAATCAAGGAGATGAG
	R:CTCGAGTTATGTCTCTTTGCCATC
**9 (304-324aa)**	F:GGATCCATGAAAGCAGCCGATCCA
	R:CTCGAGTTACTCAGCCCCGCCCTG

^a^Underlined bases indicate restriction enzymes of BamH^I^ and Xho^I^, respectively

The nine truncated genes of YB-1 were cloned into the vector, pGEX-6P-1, and identified by DNA sequencing. The YB-1 truncated peptides were expressed by the same procedures, as described above. The nine expressed truncated peptides were detected by Western blot analysis using the 1-D9 mAb, as described below.

### 3.6. SDS-PAGE and Western Blot Analysis

Proteins were separated with 10% SDS-PAGE, and then stained with Coomassie brilliant blue for 10 min at 60°C. Protein bands were observed and scanned after de-staining in solution (40% methanol, 7% acetic acid, and 53% distilled water). Following separation by SDS-PAGE, the proteins were transferred to PVDF membranes. The PVDF membranes were blocked with 1% BSA in TBS containing 0.1% Tween20 (TBST), incubated overnight at 4°C with the primary mAb (1-D9; 1:5000), YB-1 pAbs (1:500), and rabbit monoclonal anti-YB-1 (1:10000; Abcam). HRP-conjugated goat anti-mouse or anti-rabbit IgG and the ECL system were used for detection according to the manufacturer’s instructions.

### 3.7. CLIA Procedure

YB-1 in the serum sample was specifically captured by the mAb, 1-D9, which was a solid phase immobilization in microtiter wells. The recombinant YB-1 was diluted with 50% newborn calf serum to generate a series of YB-1 standards (range, 0.1-150.0 μg/L). Immunoassay procedures were performed by adding 25 μL of each sample/standards and 75 μL of HRP-conjugated pAb solutions to the microtiter wells after incubating for 1 h at 37 °C. The wells were then washed 5 times with PBST to remove unbound antibodies. A solution/well of chemiluminescent substrate (100 μL of luminol, p-iodophenol, and urea peroxide; Sigma, St. Louis, MO, USA) was then added. The mixture was incubated for 10 min at room temperature in the dark, and the RLU were read at 450 nm. The method for judging established CLIA performance, which included standard curves, linearity ranges, detection limits, recovery, intra- and inter-assay variability, and interference testing of bilirubin and hemoglobin were carried out according to the guidelines established by the Clinical Laboratory Standard Institute (22-24).

### 3.8. Detection of Serum YB-1 and AFP

The proposed CLIA was applied to quantify serum YB-1 in all enrolled subjects. AFP was also detected by an ARCHITECT i2000sr electrochemiluminescence analyzer (Abbott Diagnostics).

### 3.9. Statistical analysis

All analyses were performed with the SPSS software (version 16.0; SPSS, Inc., Chicago, IL, USA). Non-parametric data were compared using the Mann-Whitney U test. An independent samples t-test was performed to compare the means between the two groups. Significant differences were defined as a P < 0.05. Receiver operating characteristic (ROC) curve analysis for diagnosing HCC was performed to obtain the area under the ROC curve (AUC) of quantified serum YB-1 levels, and the diagnostic cut-off values.

## 4. Results

### 4.1. Expression and Purification of the YB-1 Protein

**Figure 2.**
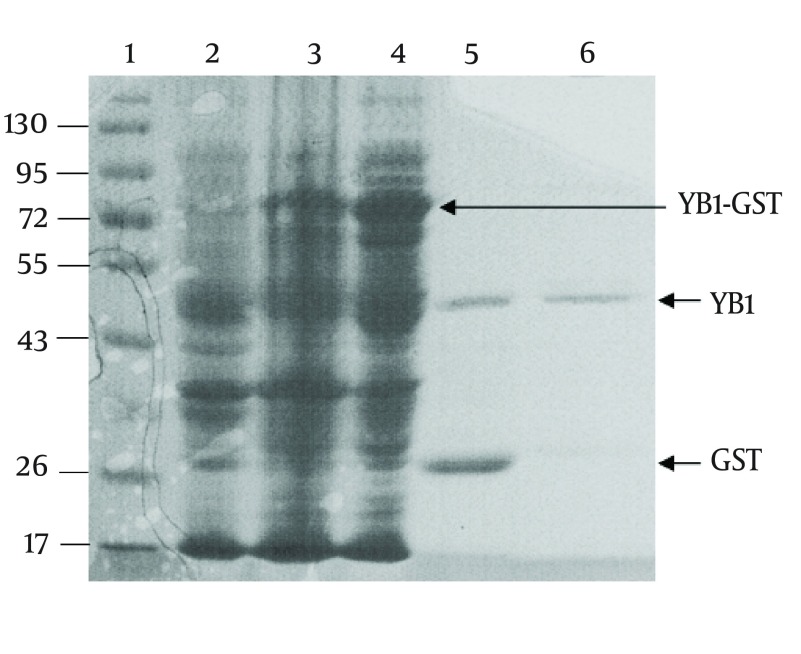
SDS-PAGE Analysis of Recombinant YB-1 Protein Expression and Purification lane 1, protein marker; lane 2, negative control; lane 3, precipitation protein; lane 4, supernatant protein; lane 5, YB1-GST digested by PSP protease; lane 6, purified recombinant YB-1 protein

The YB-1 coding sequence was obtained by RT-PCR. The coding sequence was inserted into the BamHⅠand XhoⅠ sites of the vector, pGEX-6p-1, and further confirmed by DNA sequencing (data not shown). As shown in Figure 2, the fusion protein (YB1-GST) mainly existed in the supernatants of cell lysates. Affinity purification was carried out using the GST tag of the tagged recombinant YB-1 lysates. The PSP protease recognized the GST site, and cleaved the YB1-GST with high specificity to remove the GST tag (Figure 2).

### 4.2. Characterization of the Antibodies

**Figure 3.**
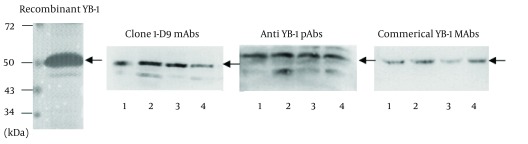
Western Blot Analysis of Prepared Antibodies Recognizing YB-1 of Recombinant Protein and Tumor Cell Lysates Recombinant YB-1 protein and lysates from tumor cells were immunostained with anti-YB-1 PAbs, mAbs 1-D9 and commercial YB-1 mAbs against YB-1. lane 1, recombinant YB-1; lane 2, breast cancer cell MDA-MB-231 lysates; lane 3, hepatic carcinoma cell HepG2 lysates; lane 4, non-small-cell lung cancer cell A549 lysates. Arrows indicated 50kDa YB-1.

The mAb, 1-D9, was of the IgG1 isotope. To identify the specificity of 1-D9 and the YB-1 pAbs, Western blot analysis was performed using a commercial YB-1 mAb (Abcam) as a reference. The band corresponding to YB-1 (50 kDa) of three cancer cell lysates (breast cancer cell, MDA-MB-231; HCC cell, HepG2; and NSCLC cell, A549) and recombinant YB-1 were recognized by the commercial YB-1 mAb, the prepared 1-D9, and the YB-1 pAbs, respectively (Figure 3).

### 4.3. The Epitope of 1-D9

**Figure 4.**
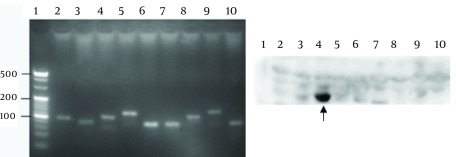
A) PCR Products of DNA Sequences 1-9 of YB-1 Lopping Peptides B) Hybridoma 1-D9 Was Capable of Recognizing the Truncated Peptide (134-160aa) in Western Blot Analysis 1, DNA ladder marker; 2-10, the DNA sequences of YB-1 lopping peptides 1-9lane 1, negative control; lane 2-10, YB-1 lopping peptides 1-9 negative control; lane 2-10, YB-1 lopping peptides 1-9

Nine truncated sequences of YB-1 were obtained by PCR using the recombinant expression vector as a template (Figure 4A), and were inserted into the vector, pGEX-6p-1. To identify the epitope recognized by 1-D9, a series of YB-1 truncated peptides were expressed. As shown in Figure 4B, 1-D9 recognized the YB-1 epitope, including the number 4 amino acids between residues 134 and 160.

### 4.4. Performance of the Proposed CLIA

**Figure 5.**
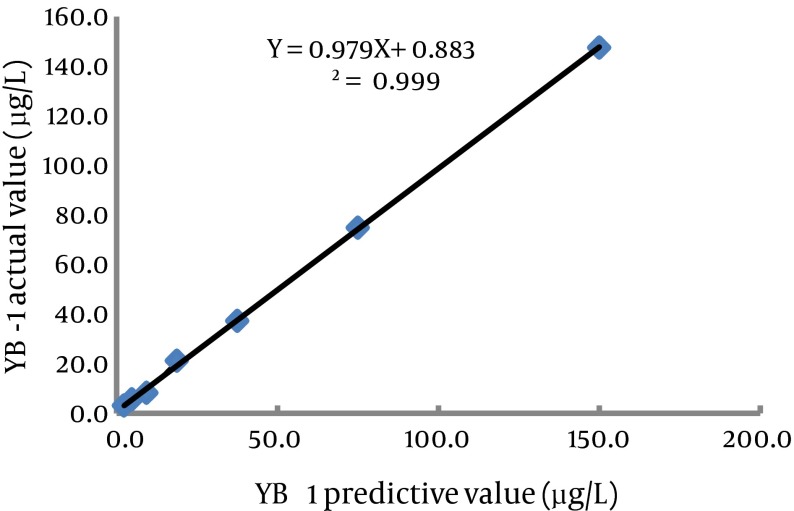
The Linearity Range of the Present Assay Serial dilution of samples with YB-1 levels of 150.0 μg/L produced good linearity of γ^2^ = 0.999

This assay used 25 μL serum samples and required 90 min to obtain results. The calibration curve (0.1-100.0 μg/L) of the CLIA had the following coefficient of linear correlation: γ2 = 0.9986 [log(Y) = 5.8178 + 0.0680log(X)]. Serial dilution of serum samples, adding YB-1 with levels of 150.0 μg/L produced linearity (γ2 = 0.999; Figure 5 ). The minimum detection limit was 0.1 μg/L (Table 3). Recovery experiments were conducted by adding 10.0 μg of recombinant YB-1 to 10 serum samples (final concentrations ranging from 13.8 to 40.7 μg/L).

**Table 3.** Minimum Limit Test of CLIA Method

YB-1, μg/L	Mean^a^	S^a^	mean-3s^a^	mean+3s^a^
**0**	5800	24.1	5727.7	5872.3
**0.1**	10009	41.5	9884.5	10133.5
**0.2**	11408	23.0	11339.0	11477.0
**0.3**	12407	32.3	12310.1	12503.9
**0.4**	13180	21.1	13116.7	13243.5
**0.5**	14360	15.9	14312.3	14407.7

^a^Relative light unit (RLU)

**Figure 6.**
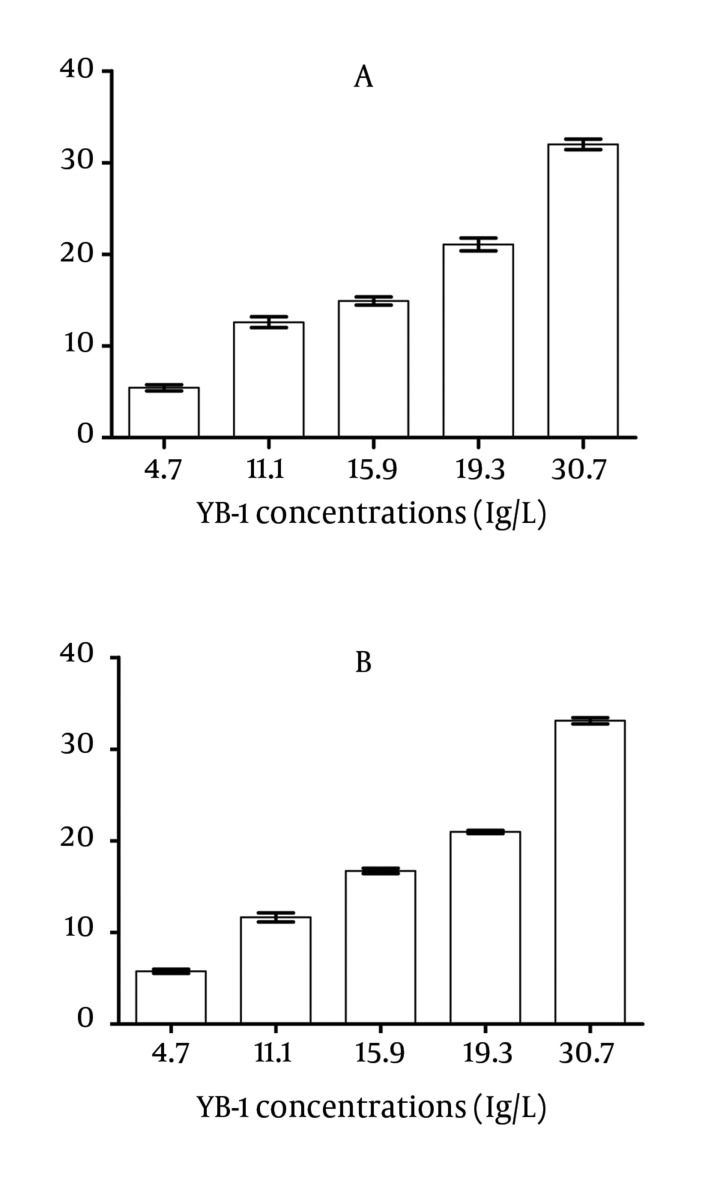
Interference Test A) Interfered by hemoglobin with 1.6, 3.2 and 6.4 g/L, respectively; B) Interfered by bilirubin with 42, 85, 171 and 342 μmol/L, respectively

The potential interference was evaluated by the addition of known increasing concentrations of hemoglobin and bilirubin. No interference was observed in the tested specimens (P > 0.05) at moderately high hemoglobin (6.4 g/L) and bilirubin (342 μmol/L) concentrations (Figure 6).

The range of recovery was 93.9%-109.0% (100.6% ± 4.7%). The intra- and inter-assay variability was examined using three pooled serum samples with average YB-1 levels of 32.9, 15.5, and 5.7 μg/L. The mean intra- and inter-assay CVs were 4.0-4.8% and 8.2-10.2%, respectively (Table 4).

**Table 4.** Inter and Intra-Assay Variability for YB-1

Concentration of Mixed Serum, μg/L	Intra-Run, n=20			Inter-Run, n=20		
	X̅	S	CV(%)	X̅	S	CV(%)
**High,32.9**	32.1	1.28	4.0	32.3	2.65	8.2
**Medium,15.5**	15.0	0.68	4.5	15.2	1.51	9.9
**Low,5.7**	5.2	0.25	4.8	5.3	0.54	10.2

### 4.5. Quantification of Serum YB-1 and Receiver Operating Characteristics (ROC)

#### 4.5.1. Serum YB-1 Concentration

**Figure 7.**
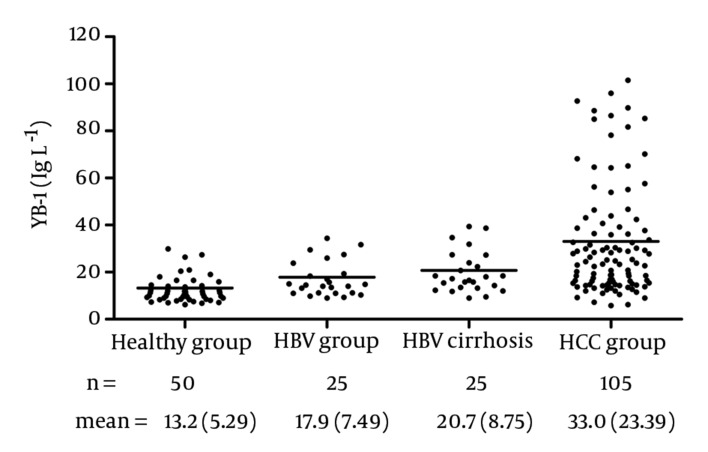
Distribution of Serum YB-1 Concentration in Various Groups The mean values were indicated by horizontal lines Abbreviations: HBV, hepatitis B virus; HCC, hepatocellular carcinoma

The YB-1 levels in the serum samples are shown in Figure 7. With this assay, the serum levels of YB-1 in HCC was 33.0 ± 23.39 μg/L, which was significantly higher than the levels in samples obtained from healthy volunteers (13.2 ± 5.29 μg/L, P < 0.0001), patients with HBV (17.9 ± 7.49 μg/L, P = 0.0003), and patients with HBV cirrhosis (20.7 ± 8.75 μg/L, P < 0.05). No relationship was observed between the serum YB-1 levels and various clinicopathologic variables, including stage, grade, and metastasis (P > 0.05).

#### 4.5.2. Sensitivity and Accuracy of YB-1 in Combination with AFP

**Figure 8.**
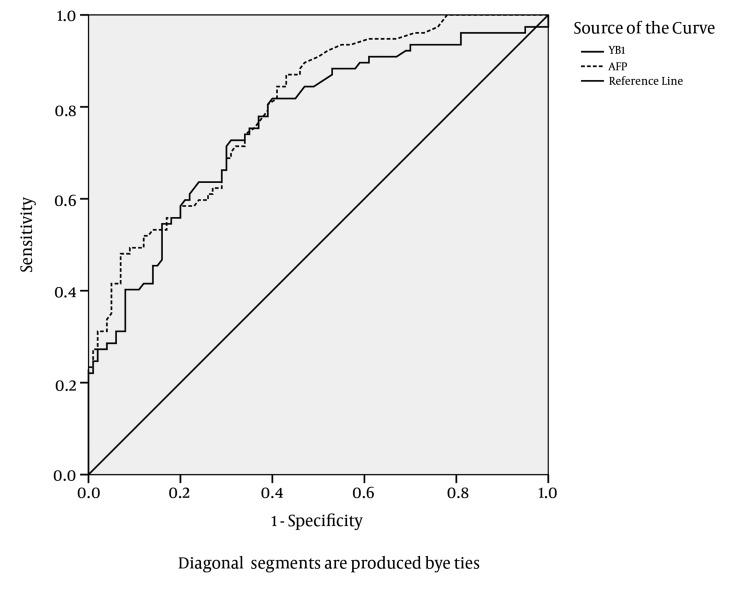
A Receiver Operating Characteristics Curve of Serum YB-1 Sensitivity vs. specificity was calculated for all healthy individuals, HBV patients, HBV cirrhosis patients and HCC patients. The graph was drawn with 50 healthy volunteers, 25 HBV patients, 25 HBV cirrhosis and 105 HCC patients.

The ROC plots of serum YB-1 for HCC diagnosis are presented in Figure 8. The area under the ROC curve (AUC) for the YB-1 curve was 0.764. The serum YB-1 had a sensitivity of 74.1% and a specificity of 63.0% at the cut-off value of 16.0 μg/L for the diagnosis of HCC. The AUC for the AFP curve was 0.798. AFP had a sensitivity of 44.8% and a specificity of 93.0% at the cut-off value of 414 μg/L. The characteristics of the diagnostic performance of serum YB-1, AFP, and YB-1+AFP for HCC are summarized in Table 5.

**Table 5.** Diagnostic Performance of Serum YB-1, AFP and YB-1+AFP for HCC

Tumor Marker	Cut-Off Value, μg/L	HCC Patients		Controls, HBV + HBV Cirrhosis + Healthy		Sensitivity, %^a^	Specificity, %^b^	Accuracy, %^c^
		Cases	Positive	Cases	Positive			
**YB-1^d^**	16.0	105	80	100	37	74.1	63.0	69.8
**AFP**	414	105	47	100	7	44.8	93.0	68.3
**YB-1+AFP**	ibid	105	94	100	38	89.5	62.0	76.1

^a^Sensitivity = TP/(TP + FN), where TP = true positive, FN = false negative

^b^Specificity = TN/(TN + FP), where TN = true negative, FP = false positive

^c^Accuracy = (TP + TN)/(TP + FP + TN + FN)

^d^Abbreviations: AFP, alpha-fetoprotein; HCC, hepatocellular carcinoma

AFP was more specific and less sensitive than YB-1 in diagnosing HCC; in contrast, serum YB-1 was more sensitive and less specific than AFP for the diagnosis of HCC. The sensitivity of YB-1 in combination with AFP for the diagnosis of HCC was improved to 89.5%. The results suggested that the serum YB-1 levels could be applied as a screening marker for HCC; especially the combination of YB-1 and AFP exhibited a good performance for screening HCC.

## 5. Discussion

Overexpressed YB-1 have been confirmed and evaluated in cancer tissues by immunohistochemistry and RT-PCR.

A previous study showed that plasma YB-1 can be detected by Western blotting (18). However, quantifying serum YB-1 has not been available so far. In our present work, the recombinant YB-1 and YB-1 pAbs and a mAb, 1-D9, were prepared. The 1-D9-detected epitope must reside within the middle domains encompassing amino acids 134-160. A CLIA for the detection of serum YB-1 was developed based on the two prepared antibodies and a double-antibody sandwich technique. The parameters of the developed method revealed that the minimum detection was 0.1 μg/L, and the within- and between-run CVs were all acceptable. By quantifying the distribution of serum YB-1 in healthy donors and patients with HCC, the cut-off value of YB-1 was first determined to be 16.0 μg/L.

Furthermore, the proposed CLIA provided a foundation for further investigation of the function and clinical significance of circulating YB-1. Major challenges of cancer in laboratory medicine relate to the diagnosis of malignant diseases and the possibility to predict the prognosis and sensitivity to chemotherapy. Regarding all these issues, the serumYB-1 may gain a prominent role, given the following: (i) YB-1 could be actively secreted (17); and (ii) extracellular YB-1 can interact with membrane receptor Notch-3 and potently up-regulate Notch target genes (25, 26) ; (iii) Notch-3 is overexpressed and promotes tumor growth in many cancers, such as HCC and lung cancer, and the suppression of Notch-3 results in loss of the malignant phenotype *in vitro *and* in vivo* (27-31); (iv) YB-1 has an oncogenic characteristic with induction of breast cancer in transgenic animals overexpressing YB-1 in the mammary gland (32); and (v) a number of reports have indicated that intracellular YB-1 up-regulates the expression of P-glycoprotein, MDR1, and the major vault protein (33-38) , and its expression levels were strongly predictive for relapse rates and negatively correlate with disease-free survival (15, 16, 34, 39-42) . In addition to the overexpression of cytoplasm YB-1 and nuclear translocalization of phosphorylated YB-1 as prognostic and chemoresistance markers in several human malignancies based on immunohistochemistry (7, 9-11, 13, 43, 44), a recent study (18) described the presence of YB-1 in the serum of patients with malignancies and healthy individuals by Western blotting, and found that YB-1 protein complexes (molecular sizes >150, 50, and 30 kDa) were present in plasma samples, including healthy donors and patients with various cancers, such as HCC and rectal cancer. Our data showed that levels of serum YB-1 in HCC was significantly higher than the 3 control groups, and had good sensitivity (74.1%) for the diagnosis of HCC. In contrast, AFP, the standard biomarker for HCC (1), had less sensitivity (44.8%), but higher specificity (93.0%). However, serum YB-1 combined with AFP showed higher sensitivity and diagnosed 89.5% of the HCC patients. It is proposed that the combination of YB-1 and AFP can be used in parallel for screening high-risk patient populations with HCC because of the good sensitivity. Negative results for YB-1 and AFP, or AFP alone, would suggest that HCC is unlikely, but a positive YB-1 (which would likely occur with a positive AFP) would make the disease highly probable. A combination of our findings and the established unique role in tumor biology suggests that serum YB-1 levels might be a potential routine tumor marker for HCC. Furthermore, the serum YB-1 quantitative assay has the advantage of being less invasive and more accurate. Because of the relative small patient number, we did not find a statistically significant correlation between the serum YB-1 levels and different stages or metastasis of HCC patients. For this purpose, a large confirmative meta-analysis on HCC cohorts will be necessary. Further investigation is valuable on whether serum YB-1 level is an aid in the diagnosis of other cancers. In conclusion, we have successfully prepared a recombinant YB-1 protein and specific antibodies. A double antibody sandwich CLIA has been developed for quantifying serum YB-1, and applied for detecting YB-1 levels in healthy individuals, and patients with HBV, HBV cirrhosis, and HCC. Our results have shown that YB-1 could distinguish patients with HCC from healthy volunteers and patients with other liver diseases. Moreover, parallel combination of YB-1 and AFP can improve the diagnostic performance compared with YB-1 or AFP alone. In addition, the YB-1 antibodies and the established CLIA method may be of importance for investigating the function of circulating YB-1, and the prospective study to validate circulating YB-1 as a chemoresistance and prognostic marker in cancer patients.
